# Physiological Responses to Supramaximal Running Exercise with End-Expiratory Breath Holding up to the Breaking Point

**DOI:** 10.5114/jhk/174465

**Published:** 2023-11-28

**Authors:** Xavier Woorons, Frédéric Daussin, Adrien Combes, Patrick Mucci

**Affiliations:** 1 URePSSS—Unité de Recherche Pluridisciplinaire Sport Santé Société, Université de Lille, Université d'Artois, Université du Littoral Côte d'Opale, Lille, France.

**Keywords:** hypoventilation, hypoxia, VHL, breath holding, muscle oxygenation

## Abstract

This study aimed to assess the physiological responses to repeated running exercise performed at supramaximal intensity and with end-expiratory breath holding (EEBH) up to the breaking point. Eight male runners participated in two running testing sessions on a motorized treadmill. In the first session, participants performed two sets of 8 repetitions at 125% of maximal aerobic velocity and with maximum EEBH. Each repetition started at the onset of EEBH and ended at its release. In the second session, participants replicated the same procedure, but with unrestricted breathing (URB). The change in cerebral and muscle oxygenation (Δ[Hb_diff_]), total haemoglobin concentration (Δ[THb]) and muscle reoxygenation were continuously assessed. End-tidal oxygen (PETO_2_) and carbon dioxide pressure (PETCO_2_), arterial oxygen saturation (SpO_2_) and heart rate (HR) were also measured throughout exercise.On average, EEBH was maintained for 10.1 ± 1 s. At the breaking point of EEBH, PETO_2_ decreased to 54.1 ± 8 mmHg, whereas PETCO_2_ increased to 74.8 ± 3.1 mmHg. At the end of repetitions, SpO_2_ (nadir values 74.9 ± 5.0 vs. 95.7 ± 0.8%) and HR were lower with EEBH than with URB. Cerebral and muscle Δ[Hb_diff_] were also lower with EEBH, whereas this condition induced higher cerebral and muscle Δ[THb] and greater muscle reoxygenation. This study showed that performing repeated bouts of supramaximal running exercises with EEBH up to the breaking point induced a fall in arterial, cerebral and muscle oxygenation compared with the URB condition. These phenomena were accompanied by increases in regional blood volume likely resulting from compensatory vasodilation to preserve oxygen delivery to the brain and muscles.

## Introduction

Training with voluntary hypoventilation at low lung volume (VHL) is a method which consists of exercising while performing short bouts of end-expiratory breath holding (EEBH). Since 2007, this method has been consistently reported to acutely induce higher levels of blood and pulmonary carbon dioxide partial pressures as well as lower blood and muscle oxygenation compared with the same exercise performed with unrestricted breathing (Ari et al., 2021; Kume et al., 2016; Toubekis et al., 2017; Woorons et al., 2007, 2010, 2014, 2017). Greater stimulation of anaerobic glycolysis, as a consequence of the hypoxic effect, has also been reported under this condition (Kume et al., 2016; Toubekis et al., 2017; Woorons et al., 2010, 2014; Zając et al., 2020). Over the last decade, the VHL approach has been acknowledged as a hypoxic method belonging to the Living Low Training High (LLTH) paradigm (Girard et al., 2017, 2020) and possibly advantageous for improving performance in many sports (Millet et al., 2010, 2019).

Until 2020, the studies that had dealt with the acute effects of VHL exercise had used EEBH of fixed duration (Kume et al., 2016; Woorons et al., 2010, 2014, 2017) or fixed distance (Woorons et al., 2019). Recently, a more ambitious approach consisting of performing EEBH up to the breaking point has been tested (Woorons et al., 2021a, 2021b). Using this approach, arterial oxygenation saturation (SpO_2_) dropped more rapidly and to a lower level than what had been reported previously. During a supramaximal cycle exercise (i.e., 150% of maximal aerobic power) performed with maximum EEBH, nadir SpO_2_ of 79% was measured and accompanied by a large increase in stroke volume and a marked drop in heart rate (Woorons et al., 2021a). SpO_2_ as low as 73% was also recorded during running bouts with maximum EEBH at 80% and 100% of the maximal aerobic velocity, provoking a large and early fall in muscle oxygenation as compared with exercise with unrestricted breathing (Woorons et al., 2021b). In those studies, EEBH was maintained on average between 9.6 and 13.2 s, depending on the exercise intensity as well as the duration and the modality (i.e., active or passive) of the recovery period.

To date, some important physiological effects of exercise with maximum EEBH remain to be investigated. First, and importantly, the impact of this specific approach on cerebral oxygenation is still unknown. A study that investigated the effects of repeated back-and-forth running sprints with non-maximum EEBH reported lower cerebral oxygenation compared with the same exercise with unrestricted breathing (Woorons et al., 2019). However, no change in cerebral blood volume was observed. This outcome may be different when EEBH is maintained for as long as possible. Furthermore, the decrease in cerebral oxygenation may be of greater magnitude under this condition. Second, the effects of exercise with maximum EEBH on muscle oxygenation have not been investigated at supramaximal intensity or at maximal velocity. Yet, it has been shown that to be effective, the LLTH approach should be performed at high exercise intensities (in particular through repeated sprints in hypoxia) to induce muscle tissue adaptations that may be favourable to performance (McLean et al., 2014). Previously, studies that used non-maximum EEBH during repeated sprints found no difference in muscle oxygenation compared with the same exercise with unrestricted breathing or, if so, only towards the end of exercise (Woorons et al., 2017, 2019). From a training perspective, it may be important to assess whether larger muscle deoxygenation may be obtained with maximum EEBH. Finally, no data are currently available regarding the levels of pulmonary partial pressures of oxygen and carbon dioxide at the breaking point of EEBH. In studies using non maximum EEBH, these variables were generally averaged over 15–20 s which could not provide their nadir and peak values. It may be interesting to determine to which point O_2_ and CO_2_ pulmonary partial pressures can drop and soar, respectively, during VHL exercise and define which of these variables is mainly involved in the breaking point of EEBH.

Within this context, the goal of the present study was to assess the physiological responses to supramaximal running exercise with maximum EEBH, in particular cerebral and muscle oxygenation through the near-infrared spectroscopy (NIRS) technique. We hypothesized that this specific exercise condition would induce lower cerebral oxygenation than during the same exercise with unrestricted breathing which should be accompanied by an increase in cerebral blood volume. We also expected larger muscle deoxygenation during most part of exercise with EEBH, possibly accentuated by a decrease in muscle blood volume, as reported during exercise with maximum EEBH performed at low and moderate intensity.

## Methods

### Participants

Eight endurance-trained male runners were recruited to participate in this study. Their physical characteristics (mean ± SD) were as follows: age 28.6 ± 8.8 years, body height 177.1 ± 3.5 cm and body mass 67.4 ± 4.7 kg. All participants were non-smokers, sea-level native and residents, and were not exposed to altitude above 500 m before participating in this study. At the time of the experiment, their regular training programme was composed of 3 to 5 sessions a week. These sessions were mainly performed at low-to-moderate intensity, but also included high-intensity exercises (90% to 110% of maximal aerobic velocity) once or twice a week. Written informed consent was provided by participants before the study which was approved by the Ethics Committee of the Lille University (protocol code 2021-483-S92; approval date: 18 May 2021) and complied with the Declaration of Helsinki (2008).

### Study Design

Participants had to attend three running testing sessions, each separated by 48 to 72 h. The sessions took place on a motorized treadmill (NordicTrack 2950, Canada) with a slope of 8%. This gradient was chosen to avoid excessive velocities during supramaximal exercises and insure the safety of participants. In the first session, participants performed a maximal incremental test to assess the maximal aerobic velocity at the slope of 8% (MAV_8%_). The test started at 6.5 km·h^−1^ and velocity was increased by 0.7 km·h^−1^ every minute until exhaustion. In the second session, participants had to perform two sets of 8 running bouts at 125% of MAV_8%_ with maximum EEBH (i.e., up to the breaking point). This specific approach of the VHL method has been used and well described in two recent studies (Woorons et al., 2021a, 2021b). Briefly, before starting each exercise bout, participants performed the first exhalation down to around the functional residual capacity, then ran on the treadmill while maintaining EEBH for as long as possible. Each bout ended at the release of EEBH (i.e., at the breaking point) with the second exhalation down to the residual volume. It was followed by a passive recovery period (standing with the feet on each side of the treadmill) of 1.5 times the duration of the previous exercise bout (work-to-rest ratio 1:1.5). Time of each bout with EEBH was measured using a stopwatch (Ultrak 495-100 Split). Both sets were separated by three minutes of passive recovery. Most of participants had taken part in the previous experiment and were therefore familiarized with the VHL technique. A verbal countdown was provided in the last 5 s before the start of each bout and strong encouragements were provided to participants to maintain EEBH for as long as possible. In the third testing session, athletes replicated exactly the same procedure as in the previous one, but with unrestricted breathing (URB).

### Measurements

#### Gas Exchange

Gas exchange was recorded continuously during the three testing sessions with a breath-by-breath portable system (K4b^2^,Cosmed, Rome, Italy). The standardized calibration procedures (air, turbine, gas and delay) were performed before each session according to the manufacturer instructions. During the first testing session, breath-by-breath measurements were performed to obtain the peak oxygen uptake (i.e., highest level of oxygen uptake averaged over a 15-s sliding window) reached during the maximal incremental test and the corresponding MAV_8%_. During the second testing session (VHL exercise), we aimed at assessing the end-tidal partial pressures in O_2_ (PETO_2_) and carbon dioxide (PETCO_2_) at the release of each EEBH, that is at the first exhalation following the end of each exercise bout, as previously done in an apnoea study (Lindholm and Linnarsson, 2002). PETO_2_ and PETCO_2_ were also assessed in the last 4 s of each recovery period following repetitions. During the third testing session (exercise with URB), PETO_2_ and PETCO_2_ were analysed in the first 4 s and the last 4 s of the recovery periods following each exercise bout.

#### Heart Rate and the Rating of Perceived Exertion

Heart rate (HR) was continuously measured during the entire exercise (*Polar* S810, *Polar*, Kempele, Finland) and the rating of perceived exertion (RPE) was evaluated at the end of each of the two sets of exercise (10-point Borg scale). *During maximum EEBH exercise, a marked drop in HR has been reported to occur in the end-phase of the breath hold periods, whereas tachycardia has been observed* during the following recovery periods (Woorons et al., 2021a, 2021b). In the present study, on the contrary, HR was expected to increase during repetitions of exercise with URB and to decrease during the recovery periods. Therefore, we aimed to target the actual nadir and peak HR reached at the end of each repetition and/or recovery period using a sliding window of 2 s.

#### Arterial Oxygen Saturation

SpO_2_ was recorded continuously (second by second) during the exercises with the pulse oximeter Nellcor PM10-N (Pleasanton, CA, USA) connected to a forehead sensor placed above the left orbital area (Max-Fast, Nellcor, Pleasanton).Data were averaged over 2-s periods to analyze the nadir values at the end or in the few seconds following the end of each repetition. Nadir SpO_2_ reached over the two sets of exercise and SpO_2_ values at the end of each recovery period were also analyzed.

#### Near-Infrared Spectroscopy

Muscle and cerebral oxygenation were evaluated through the near-infrared spectroscopy (NIRS) technique (Boushel and Piantadosi, 2000), at wavelengths between 760 and 850 nm. For muscle oxygenation, we used the PortaMon device (Artinis Medical Systems, Einsteinweg, The Netherlands) which was placed at the lower third of the left-leg vastus lateralis muscle, parallel to the long axis of the muscle and with interoptode spacing of 40 mm. Double-sided tape was utilized to firmly attach the probe to the skin. Its position was marked at the first testing session and participants were asked to keep this mark visible during the entire protocol so that the probe could be repositioned accurately at the following sessions. For cerebral oxygenation, we used the PortaLite device (Artinis Medical Systems, Einsteinweg, The Netherlands) which was positioned on the surface of the right prefrontal cortex (above the eyebrow, between the midline of the skull and the temporalis muscle) as reported in previous studies dealing with repeated sprints in hypoxia or with VHL (Billaut et al., 2013; Willis et al., 2017; Woorons et al., 2019). Double sided tape was also utilized to attach the probe and a head wrap was added to create a dark environment and maintain a stable position. For the vastus lateralis muscle, we used a differential pathlength factor of 4.0, whereas for the prefrontal cortex we used an age-dependent one (Duncan et al., 1996). All NIRS signals were recorded with a sampling frequency of 10 Hz and a 10^th^-order low-pass zero-phase Butterworth filter (cut-off frequency of 0.1 Hz) was applied to reduce artefacts and smooth the perturbations in the signal (Woorons et al., 2019).

Concentrations of oxyhaemoglobin ([O_2_Hb]) and deoxyhaemoglobin ([HHb]) were recorded at the cerebral level, while muscle oxy-haemoglobin/myoglobin ([O_2_Hb/Mb]) and deoxy-haemoglobin/myoglobin ([HHb/Mb]) concentrations were measured with the Portamon device. The sums ([THb], [THb/Mb]) of [O_2_Hb] and [HHb] and of [O_2_Hb/Mb] and [HHb/Mb] were calculated to obtain cerebral total haemoglobin and muscle total haemoglobin/myoglobin, respectively, which were used as indices of regional blood volume. To analyse cerebral and muscle oxygenation, we calculated the difference ([Hb]_diff_, [Hb/Mb]_diff_) between [O_2_Hb] and [HHb] and between [O_2_Hb/Mb] and [HHb/Mb], respectively. These indices have been used for long (Grassi et al., 2003; MacDonald et al., 1999) and are considered reliable and relevant, in particular when total haemoglobin is not constant (van Beekvelt et al., 2002). In the lead-in period of the present study, it is important to note that large variations of both cerebral [THb] and muscle [THb/Mb] were observed during running exercises at 125% of VAM_8%_ and during the following recovery periods. With regard to these observations, and considering that greater change in [THb] and [THb/Mb] was expected during VHL exercise, the use of [O_2_Hb] and [HHb] or [O_2_Hb/Mb] and [HHb/Mb] for assessing both cerebral and muscle oxygenation may have biased the results. It was therefore more relevant to use [Hb]_diff_ and [Hb/Mb]_diff_. For the two sets of exercise, changes (Δ) in cerebral [THb] and [Hb]_diff_ and in muscle [THb/Mb] and [Hb/Mb]_diff_were assessed from the resting values (seated position) recorded over the 3 min preceding the start of the test. The measurements were therefore normalized from these recordings (arbitrarily defined as 0 µm).

For all variables, data were averaged over 2 s and a rolling approach was used to obtain the peak values of cerebral Δ[THb] and the nadir values of cerebral Δ[Hb]_diff_, muscle Δ[THb/Mb] and muscle Δ[Hb/Mb]_diff_at the end or in the few seconds following each repetition, as observed in the lead-in period of the study. We also analysed all of these variables at the end of each recovery period. To assess muscle reoxygenation capacity (Reoxy[THb/Mb], Reoxy[Hb/Mb]_diff_), we calculated the difference in Δ[THb/Mb] and in Δ[Hb/Mb]_diff_ between the end of each recovery period and the end of the previous repetition (Billaut and Buchheit, 2013).

### Statistical Analysis

Before performing the analyses, distribution normality and variance homogeneity of data were tested. We then performed Student *t*-tests to determine whether there was a difference in the mean values of all variables between exercise conditions over the two sets of exercise. Two-way repeated measures ANOVA was also performed to assess whether there was a difference in variablesbetween conditions (only) for each repetition and the recovery period following repetitions (averaged over the two sets). When a significant effect was found, the Bonferroni post-hoc test was carried out to localize the differences.All analyses were performed using Sigmastat 4.0 software (Systat Software, CA, USA). All results are expressed as mean ± SD and we rejected the null hypothesis at *p*< 0.05.

## Results

The results of the maximal incremental test are displayed in Table 1. All participants could complete all the repetitions of the two sets of VHL exercise. On average, EEBH was maintained for 10.1 ± 1.0 s.

**Table 1:** Results of the maximal incremental test.

Subjects n = 8	
	Mean ± SD
MAV_8%_ (km·h^−1^)	14.7 ± 0.8
${\dot{\text{V}}\text{O}}_{2}$ peak (ml·min^−1^·kg^−1^)	63.5 ± 4.5
Heart rate peak (bpm)	187.1 ± 5.4
Nadir SpO_2_ (%)	93.2 ± 2.0
End-exercise cerebral Δ[Hb]_Diff_ (µm)	0.4 ± 1.1
End-exercise cerebral Δ[THb] (µm)	10.7 ± 9.6
End-exercise muscle Δ[Hb/Mb]_Diff_ (µm)	−36.3 ± 14.2
End-exercise muscle Δ[THb/Mb] (µm)	9.4 ± 12.9
Rating of perceived exertion	9.0 ± 0.3

MAV_8%_, maximal aerobic velocity at a 8% treadmill slope; ${\dot{\text{V}}\text{O}}_{2}$

, oxygen uptake; SpO_2_, arterial oxygen saturation; Δ[Hb]_Diff_, change in haemoglobin oxygenation; Δ[THb], change in total haemoglobin;Δ[Hb/Mb]_Diff_, change in haemoglobin/myoglobin oxygenation; Δ[THb/Mb], change in total haemoglobin/myoglobin.

### Gas Exchange

**Figure 1:**
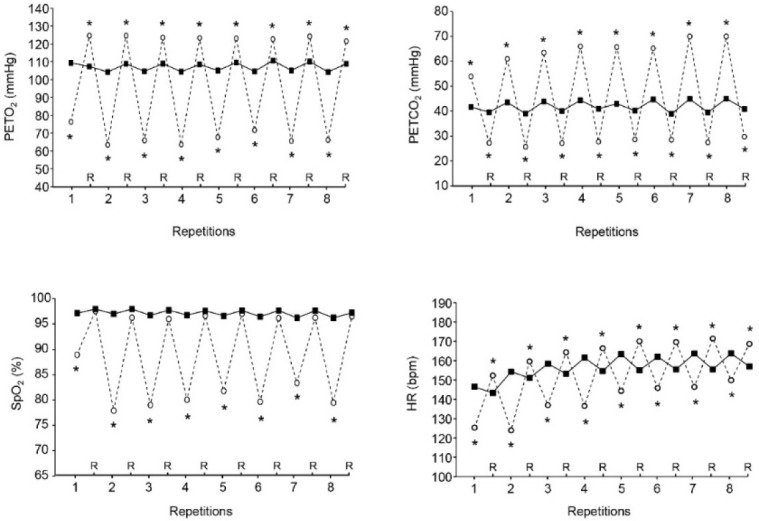
Mean end-tidal partial pressures in oxygen (PETO_2_, upper left panel), end-tidal partial pressures in carbon dioxide (PETCO_2_, upper right panel), arterial oxygen saturation (SpO_2_, lower left panel) and heart rate (HR, lower right panel) at the end of each repetition and recovery period (R) over the two sets of exercise with voluntary hypoventilation at low lung volume (dashed lines) and with unrestricted breathing (URB, solid line). *, significant difference with URB.

In VHL, mean and nadir PETO_2_ and mean and peak PETCO_2_ at the release of EEBH were lower and higher, respectively, than the values recorded in URB at the end of repetitions over the two sets of exercise (Table 2). Furthermore, compared with URB, PETO_2_ was lower at the end of each repetition in VHL and higher at the end of each recovery period (Figure 1). Conversely, PETCO_2_ was higher in VHL at the end of all repetitions and lower at the end of all recovery periods.

**Table 2:** PETO_2_, PETCO_2_, SpO_2_ and HR values over the two sets of exercise with VHL and URB.

	VHL	URB
Mean end-repetition PETO_2_ (mmHg)	67.6 ± 4.4 ^*^	105 ± 1.7
Mean end-recovery PETO_2_ (mmHg)	123 ± 1.1 ^*^	109 ± 1.0
Nadir PETO_2_ of exercise	54.1 ± 8.1 ^*^	102 ± 2.8
Mean end-repetition PETCO_2_ (mmHg)	64.4 ± 5.2 ^*^	43.9 ± 1.2
Mean end-recovery PETCO_2_ (mmHg)	27.8 ± 1.2 ^*^	39.9 ± 0.8
Peak PETCO_2_ of exercise (mmHg)	74.8 ± 3.1 ^*^	45.1± 2.1
Mean end-repetition SpO_2_ (%)	81.3 ± 3.7 ^*^	96.7 ± 0.8
Mean end-recovery SpO_2_ (%)	96.5 ± 0.6 ^*^	97.7 ± 0.3
Nadir SpO_2_ of exercise (%)	74.6 ± 3.4 ^*^	95.7 ± 0.7
Mean end-repetition HR (bpm)	138 ± 12 ^*^	160 ± 10
Mean end-recovery HR (bpm)	165 ± 11 ^*^	153 ± 10

Values are mean ± SD. PETO_2_, end-tidal oxygen partial pressure; PETCO_2_, end-tidal carbon dioxide partial pressure; SpO_2_, arterial oxygen saturation; HR, heart rate; VHL, voluntary hypoventilation at low lung volume; URB, unrestricted breathing; *, significantly different from URB; *p*< 0.01.

### Arterial Oxygen Saturation

Mean end-repetition SpO_2_, mean end-recovery SpO_2_ and nadir SpO_2_ over the two sets of exercise were lower in VHL than in URB (Table 2). Figure 1, which displays the kinetics of SpO_2_ throughout an entire set, shows that it was also lower in VHL at the end of each repetition, whereas no difference was observed at the end of the recovery periods.

### Heart Rate and the Rating of Perceived Exertion

Both the mean end-repetition and end-recovery HR were respectively lower and higher in VHL than in URB (Table 2). Figure 1 shows that HR was lower at the end of each repetition and higher at the end of all recovery periods in VHL than in URB. The mean RPE at the end of the sets was higher in VHL than in URB (9.1 ± 0.8 vs. 7.7 ± 0.7, *p*< 0.01).

### NIRS Data

On average, at the end of the repetitions of both sets, cerebral Δ[THb] and muscle Δ[THb/Mb] were higher in VHL than in URB, whereas cerebral Δ[Hb]_diff_ and muscle Δ[Hb/Mb]_diff_ were lower in VHL (Table 3). At the end of the recovery periods following repetitions, mean cerebral Δ[THb] and Δ[Hb]_diff_ as well as mean muscle Δ[THb/Mb] were also higher in VHL than in URB, whereas mean muscle Δ[Hb/Mb]_diff_ was lower in VHL. Muscle Reoxy[THb/Mb] and Reoxy[Hb/Mb]_Diff_ were greater in VHL than in URB.

**Table 3:** Mean NIRS values over the two sets of exercise with VHL and URB.

	VHL	URB
Cerebral		
End repetition		
Δ[THb] (µm)	18.1 ± 7.9 ^*^	7.6 ± 5.8
Δ[Hb]_Diff_ (µm)	−4.4 ± 3.7 ^*^	−1.6 ± 3.5
End recovery		
Δ[THb] (µm)	9.4 ± 5.9 ^*^	1.5 ± 3.6
Δ[Hb]_Diff_ (µm)	3.3 ± 2.8 ^*^	1.9 ± 3.3
Muscle		
End repetition		
Δ[THb/Mb] (µm)	−0.7 ± 6.6 ^*^	−6.0 ± 3.6
Δ[Hb/Mb]_Diff_ (µm)	−38.4 ± 13 ^*^	−23.8 ± 8.0
End recovery		
Δ[THb/Mb] (µm)	25.1 ± 8.3 ^*^	11.5 ± 6.4
Δ[Hb/Mb]_Diff_ (µm)	−12.8 ± 7.8 ^*^	−8.8 ± 8.0
End-repetition-to-end-recovery reoxygenation		
Reoxy[THb/Mb] (µm)	26.2 ± 14 ^*^	17.5 ± 5.7
Reoxy[Hb/Mb]_Diff_ (µm)	25.6 ± 14 ^*^	15.1 ± 10.9

Values are mean ± SD. NIRS, near-infrared spectroscopy; VHL, voluntary hypoventilation at low lung volume; URB, unrestricted breathing; Δ[THb], change in total haemoglobin concentration; Δ[Hb]_Diff_, change in haemoglobin oxygenation; Δ[THb/Mb], change in total haemoglobin/myoglobin concentration; Δ[Hb/Mb]_Diff_, change in haemoglobin/myoglobin oxygenation; Reoxy[THb/Mb] (µm), change in total haemoglobin/myoglobin concentration during the recovery periods; Reoxy[Hb/Mb]_Diff_, haemoglobin/myoglobin reoxygenation. *, significantly different from URB; *p*< 0.01.

**Figure 2:**
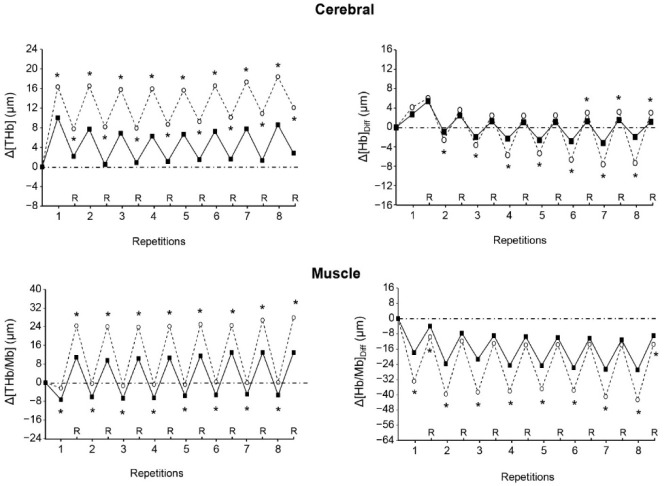
Changes in the mean cerebral total haemogolin (Δ[THb], upper left panel), cerebral oxygenation (Δ[Hb]_Diff_, upper right panel), muscle total haemoglobin/myoglobin (Δ[THb/Mb], lower left panel) and muscle oxygenation (Δ[Hb/Mb]_Diff_ , lower right panel) at the end of each repetition and recovery period (R) over the two sets of exercise with voluntary hypoventilation at low lung volume (dashed lines) and with unrestricted breathing (URB, solid line). *, significant difference with URB.

Figure 2 shows that both cerebral Δ[THb] and muscle Δ[THb/Mb] were higher in VHL than in URB at each repetition and recovery period of exercise. Muscle Δ[Hb/Mb]_diff_ was lower in VHL at the end of each repetition, but was mostly not different between conditions at the end of the recovery periods. Finally, cerebral Δ[Hb]_diff_ was lower in VHL at the end of most repetitions, whereas it was not different or higher as compared with URB at the end of the recovery periods.

## Discussion

To the best of our knowledge, this study is the first to investigate the physiological responses to repeated bouts of supramaximal running exercises (i.e., 125% of VAM_8%_) performed with EEBH up to the breaking point. The main result is that this condition induced lower cerebral oxygenation and a fall in muscle oxygenation as compared with the URB condition, which was likely the consequence of the marked and early drop in SpO_2_. An original outcome was that these phenomena were accompanied by an increase in regional blood volume both at the cerebral and the muscle level. Another interesting and original result is the greater muscle reoxygenation that occurred during the recovery periods following the exercise bouts with maximum EEBH.

Several studies have already investigated the effects of VHL exercise on muscle oxygenation using different exercise intensities (Kume et al., 2016; Woorons et al., 2010, 2017, 2019). However, as far as we know, only one used EEBH up to the breaking point (Woorons et al., 2021b). Under this condition, a large and early decrease in muscle oxygenation was reported during repeated bouts of exercise at 60, 80 and 100% of MAV, as compared with the same exercise with unrestricted breathing. The lower muscle Δ[Hb/Mb]_diff_ observed in VHL in the present study from the beginning to the end of exercise demonstrates that such results may also be obtained at supramaximal intensity. Previously, it has been shown that the use of non-maximum EEBH during repeated-sprint exercises provoked only limited effects on muscle oxygenation (Woorons et al., 2017, 2019). It is noteworthy that EEBH maintained up to the breaking point during the repeated bouts at 125% of VAM_8%_ induced a much larger drop in SpO_2_ than what has been reported in the above mentioned studies which used EEBH of ~6 s (nadir SpO_2_ values: 75% vs. 85–86%). This large drop in arterial oxygenation probably led to greater muscle venous oxygen desaturation in order to try and maintain the arterio-venous O_2_ difference and, possibly, to lower myoglobin concentration.

While muscle oxygenation was significantly lower at the end of all repetitions of exercise with VHL, it was mostly not different between conditions at the end of the recovery periods (Figure 2). This result is probably due to the rise in SpO_2_ during the recovery periods following the exercise bouts with maximum EEBH (Figure 1), as a consequence of the resumption of breathing and the resulting hyperventilation. Thus, unlike an exercise carried out in an hypoxic environment, the hypoxic stimulus is not continuous during VHL exercise. Although SpO_2_ levels reported in the present study and the previous one (Woorons et al., 2021a) show that the degree of hypoxia during exercise bouts with maximum EEBH is as severe as during high-intensity exercise performed at simulated altitudes of 3500–3800 m (Bowtell et al., 2014; Willis et al., 2017), the hypoxic effect only lasts few seconds at each repetition. Whether this feature may lead to different physiological adaptations as after a period of LLTH carried out in normobaric or hypobaric hypoxia remains to be investigated.

The greater Δ[THb/Mb] that accompanied the lower Δ[Hb/Mb]_diff_at the muscle level during the exercise bouts with EEBH represents a novel finding which was not expected and contradicts our hypothesis. In the previous VHL study using maximum EEBH, lower muscle Δ[THb/Mb] was reported compared with exercise with unrestricted breathing (Woorons et al., 2021a). The authors hypothesized that this may have been caused by peripheral vasoconstriction at the muscle tissue level to counteract the large decrease in blood oxygenation and protect the brain from hypoxia. A certain amount of blood could thus be redirected to the brain and insure an adequate O_2_ supply. In this study, however, it is noteworthy that the lower Δ[THb/Mb] was recorded only during the first four bouts of exercise at 100% of MAV, while it was observed in all the exercise bouts with maximum EEBH at 60% and 80% of MAV. The authors assumed that during high-intensity exercise, when the need for O_2_ increases, vasoconstriction of the active muscles may not occur because it could severely impair their O_2_ perfusion. The present outcomes reinforce this assumption and even tend to show that when exercise intensity is supramaximal, muscle vasodilation would be necessary if not to counteract at least to limit the decrease in O_2_ perfusion under the effect of maximum EEBH.

Remarkably, during the recovery periods, the greater muscle Reoxy[THb/Mb] in VHL, which led to high Δ[THb/Mb] compared to the resting levels and accentuated the magnitude of the difference in this variable between conditions (Figure 2, Table 3), shows that a large amount of blood was redirected to the muscle. It is therefore likely that strong muscle vasodilatation occurred, which may have been mediated, at least in part, by the large increase in hydrogen ions (H^+^) under the effect of both hypoxia and hypercapnia. Interestingly, during exercise in hypoxia, compensatory muscle vasodilation has been evoked to preserve muscle O_2_ delivery (Casey and Joyner, 2012). It has been shown that the release in nitric oxide could play an important role in this compensatory vasodilation (Casey et al., 2010; Wilkins et al., 2008). Therefore, it is possible that nitric oxide-mediated vasodilation mechanisms may have also played a role in larger Δ[THb/Mb] in the VHL condition. The fact that such a finding was not reported in the previous VHL study shows that the use of supramaximal intensities or maximal velocities, rather than low or moderate exercise intensities, is paramount to induce muscle vasodilation.

Another important finding of this study, which has not been reported previously, is the large increase in regional blood volume observed at the cerebral level at the end of each repetition with EEBH (Figure 2). It supports the hypothesis that a certain amount of blood has to be redirected to the brain to protect it from hypoxia when exercise is performed with maximum EEBH. Previously, despite lower cerebral oxygenation, no change in cerebral blood volume was found during repeated 6-s sprints with EEBH compared with sprints with unrestricted breathing (Woorons et al., 2019). Again, it is likely that the use of non-maximum EEBH during exercise does not provoke a sufficiently strong hypoxic stimulus to induce a significant compensatory increase in cerebral blood volume. Conversely, the marked hypoxic effect induced by EEBH up to the breaking point, which led to a fall in cerebral Δ[Hb]_diff_ , may have activated mechanisms leading to cerebral vasodilation and therefore to an augmented blood volume. Hypoxia has been reported to stimulate the production of many vasodilator metabolites, among which H^+^, potassium ions, prostaglandins and adenosine play the major role (Pearce, 1995). Importantly, hypoxia-induced cerebral vasodilation was likely reinforced by the strong hypercapnic effect provoked by the maximum EEBH, as demonstrated by the high levels of PETCO_2_. Pryblowski et al. (2003) showed that hypercapnia, and the consecutive increase in H^+^, could be the primary cause of vasodilation of the cerebral arterioles during apnoea at rest. Thus, in the present study, both the strong hypoxic and hypercapnic effects probably contributed to the dramatic increase in cerebral blood volume at the end of repetitions with EEBH.

It is surprising to note that cerebral Δ[THb] remained higher in VHL at the end of the recovery periods following repetitions, whereas, in the same time, PETO_2_ and PETCO_2_ were respectively higher and lower than in URB and SpO_2_ was not different between conditions. This outcome may be explained by the fact H^+^ concentrations were probably still high at the cessation of exercise. It is well known that H^+^ continues to increase over the few minutes following intense exercise (Stringer et al., 1992). Thus, despite a transient hypocapnic and normoxic state during the recovery periods of VHL exercise, it is likely that the high levels of acidosis continued to provoke cerebral vasodilation, similar to what was observed at the muscle level.

The increase in both cerebral and muscle blood volume that occurred at the end of each repetition with EEBH probably led to an upregulation of the local blood flow and, in turn, of cardiac output. Yet, it is noteworthy that, as previously reported (Woorons et al., 2021a, 2021b), there was a marked drop in HR at the end of the repetitions with EEBH compared with the URB condition. As a consequence, a large compensatory increase in stroke volume should have occurred to insure adequate perfusion of the brain and the active muscles. This would confirm the finding of Woorons et al. (2021a) who reported an overshoot of the left ventricular stroke volume during repeated bouts of supramaximal cycle exercises with maximum EEBH, leading to augmented cardiac output.

As far as we know, this study is the first to provide data relative to the levels of PETO_2_ and PETCO_2_ at the breaking point of EEBH during repeated bouts of exercise. Previously, a study dealing with the effects of apnoea in exercising men reported much lower PETO_2_ (nadir values: 38 mmHg vs. 54 mmHg), but also lower PETCO_2_ (peak values: 65 mmHg vs.75 mmHg) than in our study (Lindholm and Linnarsson, 2002). However, these data are difficult to compare to the present ones considering that the conditions were quite different. Indeed, those authors used a succession of single apnoea each separated by several minutes of eupnoea and which were performed at high lung volume and during a cycle exercise at low intensity (i.e., 120 W). Yet, based on the data reported in that previous study and those of the present one, in particular the high levels of PETCO_2_, it may be postulated that hypercapnia could have been the primary cause of the breaking point of EEBH during VHL exercise.

A first limitation that could be considered in the present study is that both cerebral (CBF) and muscle blood flow were not measured. However, techniques such as Xenon clearance, single-photon/positron emission tomography or functional magnetic resonance for CBF measurement on the one hand, and thermodilution, ultra sound doppler or plethysmography for measuring muscle blood flow on the other, are difficult to implement during running exercises, in particular at high intensity (Rådegran, 1999). Furthermore, with such techniques, measurements generally take a certain time to be performed and/or cannot provide continuous recording. Yet, this is problematic with VHL exercise which induces large and fast changes in physiological responses. Therefore, these techniques would not have enabled to determine the actual differences in the local blood flow with URB. On the other hand, the use of the NIRS technique seems relevant considering that the change in NIRS data has a very similar time course to CBF when measured by transcranial Doppler ultrasound (Hirth et al., 1997). We could thus confidently assert that both the cerebral and the muscle blood flow were significantly increased during VHL exercise in the present study. One may also argue that the measurement of cerebral oxygenation at the right prefrontal cortex only could represent another limitation of this study. However, during VHL exercise, physiological effects are systemic rather than local. It is therefore highly unlikely that the other parts of the brain may have been differentially oxygenated. Furthermore, the measurement at the prefrontal site may be relevant since it has been suggested to contribute to fatigue during exercise (Subudhi et al., 2007). Finally, the volume of the first exhalation after each EEBH was not controlled in this study. While it probably differed between participants considering that their physical characteristics were not similar, it is unlikely that it was different within subjects. Indeed, we carefully ensured that all the participants respected the breathing procedure during the VHL condition. Therefore, this point may not have negatively affected the results.

The results of the present study may have interesting implications for training optimization and performance enhancement in athletes. First, it has been shown that the LLTH approach, to which the VHL method can be assimilated (Girard et al., 2017, 2020), is particularly effective when using exercises at high intensities or maximal velocities rather than low or moderate intensities (McLean et al., 2014). Therefore, performing repeated bouts of supramaximal exercise with maximal EEBH may allow athletes to benefit from intermittent hypoxic training without going to or simulating altitude through expensive devices. Second, the compensatory muscle vasodilation that probably occurs during this kind of exercise is important in a training perspective since physiological adaptations leading to improved muscle perfusion could be expected after several weeks of such a training regimen, as reported after a period of repeated-sprint training in hypoxia (Faiss et al., 2013, 2015). Higher blood perfusion at the muscular level is paramount for improving the repeated-sprint ability (Girard et al., 2017). It is, however, important to mention that the VHL approach, as performed in its extreme form, should be used with caution considering its demanding feature and the large drop in oxygen levels it induces.

In summary, this study showed that performing repeated bouts of supramaximal running exercise with EEBH up to the breaking point induced a large drop in arterial oxygen saturation, leading to an early and marked fall in muscle oxygenation as well as a decrease in cerebral oxygenation compared with the same exercise with unrestricted breathing. The VHL condition also led to an augmented regional blood volume which was observed both at the cerebral and the muscle level. One could assume that this phenomenon reveals compensatory vasodilation in order to preserve oxygen delivery to the brain and muscles. This may have been the consequence not only of the hypoxic effect, but also the strong hypercapnic effect induced by the VHL condition, as shown by the high levels of PETCO_2_ at the breaking point of EEBH.
